# Bevacizumab for treating Hereditary Hemorrhagic Telangiectasia patients with severe hepatic involvement or refractory anemia

**DOI:** 10.1371/journal.pone.0228486

**Published:** 2020-02-07

**Authors:** Carolina Vázquez, María Laura Gonzalez, Augusto Ferraris, Juan Carlos Bandi, Marcelo Martín Serra

**Affiliations:** 1 A.R.G Argentine Rendu Study Group, Buenos Aires, Argentina; 2 Internal Medicine Department, Hospital Italiano, Buenos Aires, Argentina; 3 Hereditary Hemorrhagic Telangiectasia Unit, Hospital Italiano, Buenos Aires, Argentina; 4 Gastroenterology Department, Hospital Italiano, Buenos Aires, Argentina; 5 Hepatology Department, Hospital Italiano, Buenos Aires, Argentina; Medizinische Fakultat der RWTH Aachen, GERMANY

## Abstract

**Objective:**

To report our clinical experience with bevacizumab in a cohort of Hereditary Hemorrhagic Telangiectasia (HHT) patients with severe hepatic involvement and/or refractory anemia.

**Methods:**

Observational, ambispective study of the Institutional Registry of HHT at Hospital Italiano de Buenos Aires. Patients were treated with bevacizumab due to iron deficiency refractory anemia secondary to nasal/gastrointestinal bleeding and/or high output cardiac failure. We describe basal clinical data, bevacizumab schedules, efficacy outcomes and adverse events. Wilcoxon signed ranks test and longitudinal analysis were conducted.

**Results:**

Twenty adult patients were included from July 2013 to June 2019. Clinical indications were: 13 for anemia, 4 for heart failure and 3 for both. In the anemia group, median pretreatment hemoglobin was 8.1 g/dl [IQR: 7.2–8.4] and median transfusion requirement was 4 units [2–6]. In heart failure group, pretreatment median cardiac index was 4.5 L/min/m^2^ [4.1–5.6] and cardiac output was 8.3 L/min [7.5–9.2]. Bevacizumab 5 mg/kg/dose every 2 weeks for 6 applications was scheduled. By the end of induction, median hemoglobin at 3 months was 10.9 g/dl [9.5–12.8] (*p* = 0.01) and median transfusion requirement 0 units [0–1] (*p*<0.01), and this effect was more or less sustained during a year. Regarding heart failure group, two patients had complete hemodynamic response and achieved liver transplantation and two had partial response. No serious adverse events were registered.

**Conclusion:**

Bevacizumab is a promising line of treatment for HHT patients with refractory anemia. For patients with high output cardiac failure, bevacizumab may be useful as bridge therapy awaiting for liver transplantation.

## Introduction

Hereditary Hemorrhagic Telangiectasia (HHT) is an autosomal dominant vascular dysplasia caused by mutations in genes involved in the TGF*β-*BMP receptor signaling pathway: *ENG* (HHT type 1), *ACVRL1* (HHT type 2) and *MADH4* (HHT overlap syndrome with Juvenile Polyposis) [1]. HHT is a rare condition that affects around 1/5000 individuals worldwide [2,3]. Mucocutaneous telangiectasias in typical sites like face, lips, hands, digestive tract and vascular malformations (VMs) affecting internal organs such as brain (CAVMs), lungs (PAVMs), and liver (HVMs) are hallmarks of HHT. The diagnosis is based on Curazao Criteria [4] and/or genetic testing. Nasal telangiectasias cause epistaxis in 95% of cases which can lead to chronic iron deficiency anemia or serious acute life threatening episodes. Gastrointestinal (GI) telangiectasias are usually present in about 80% of patients with HHT, but only one third of these suffer from symptoms. GI bleeding usually starts in the fifth decade of life, mainly in women and the most common presentation is occult hemorrhage [5]. Anemia may develop in at least half of the patients and some of them could become refractory to intensive iron, endoscopic or transfusion therapy. Regarding hepatic involvement, eighty percent of HHT patients may harbor HVMs, especially in HHT type 2, though only 8% of them suffer symptoms according to the predominant shunt established [6]. The most common presentation of the HVMs is high output cardiac failure (HOCF) with secondary pulmonary hypertension due to arteriovenous malformations. Other HVMs include arterio-portal or veno-portal shunt, leading to portal hypertension syndrome with or without hepatic encephalopathy or biliary tree ischemic necrosis [7].

At the moment, there is no cure for this disease. However, there are therapies in development including pharmacological approach and intervention/surgical procedures [8]. Drugs used to treat nose and GI bleeding include: antifibrinolytics [9,10], hormone therapy [11], immunosuppressant [12,13] and antiangiogenics [14–17]. The initial management of symptomatic HVMs include: diuretics, antiarrhythmic and cardiotonics drugs for treating HOCF and/or classical portal hypertension treatment while ischemic cholangitis management include antibiotics and clinical support. Nevertheless some patients with severe hepatic disease could become refractory to these treatments, needing liver transplantation (LT)[18,19]. The embolization of liver VMs should be avoided as it is associated with biliary ischemic necrosis and high mortality [20].

Altered angiogenesis associated with HHT, is a complex biological abnormal process in which the vascular endothelial growth factor (VEGF) is elevated [21,22]. Hence, increasing interest in the role of antibodies targeting VEGF for the treatment of severe forms of HHT has arisen, especially for those patients with iron deficiency refractory anemia (IDRA) and/or progressive hepatic disease leading to HOCF, as a bridge to LT or for cases in which LT is contraindicated or unaccepted [23]. Bevacizumab, a humanized antibody against VEGF approved for the treatment of many types of cancer has been used for treating patients with severe forms of HHT. Though it is still prescribed as an off label drug, mounting scientific evidence shows its efficacy and safety [24–27].

Our goal is to describe and analyze efficacy and adverse outcomes in patients with HHT who begin bevacizumab either by HOCF and/or IDRA.

## Patients and methods

An ambispective observational study was designed and conducted at HHT Referral Center of Hospital Italiano de Buenos Aires, Argentina. Patients were eligible for enrollment if they were older than 18 years old, had definite HHT based on the presence of 3 or more Curazao criteria and/or confirmed genetic testing; and had initiated treatment with intravenous bevacizumab (reference or bio similar drug was used interchangeably) either because of having refractory HOCF and/or IDRA. Data was obtained during June/July 2019 from the Institutional HHT Registry (ClinicalTrials.gov Identifier: NCT0176198) which was started on 2012. This registry has been approved by the local ethics review board, and each included patient has consented to the use of data for research purposes. All the data analyzed in this study were properly anonymized.

### Baseline measured variables and definitions

Cases of refractory HOCF were defined as patients with chronic heart failure class III/IV according to the classification of the New York Cardiac Association, despite adequate hydrosaline restriction and medical therapy with diuretics and beta blockers. IDRA cases were defined as those patients with hemoglobin levels persistently below 10 g/dl despite adequate iron replacement therapy (according to Ganzoni formula)[28] and despite adequate transfusion support. Intravenous iron was usually preferred over transfusion support with red blood cells, except in those cases of hemoglobin less than 7 g/dl or above this value but with overt active bleeding, persistent occult bleeding, highly symptomatic anemia and/or severe comorbidities.

Demographic characteristics, comorbidities and available analytical data were recorded for each enrolled patient and extracted from both clinical interviews and medical records. For HOCF patients, pretreatment cardiac index (CI) in L/min/m^2^ and cardiac output (CO) in L/min by echocardiography and/or cardiac catheterization when possible, were recorded. For IDRA cases, due to high variability of hemoglobin values in short periods of time, for each separate patient, *baseline* hemoglobin level (g/dl) was defined as the average of all recorded values up to three months before starting treatment. Total amount of red blood cell units and intravenous iron (mg) received up to three months before treatment were also registered. Regarding endoscopic argon plasma coagulation (APC) procedures, total number of therapeutic endoscopies in life and *baseline* were recorded, the latter defined as number of APC procedures up to three months before starting bevacizumab. The epistaxis severity was recorded through the Epistaxis Severity Score (ESS) [29] before starting treatment.

Standard bevacizumab induction schedule was defined as receiving 5 mg/kg every 14 days for a total of 6 applications in a bevacizumab-naive patient. Re-induction schedule was defined as receiving 5 mg/kg every 14 days for a total of 6 applications after having received a complete induction schedule; and maintenance schedule was defined as receiving 5 mg/kg every variable periods of time such as every one, two or three months for a variable number of applications depending on clinical response. The clinical criteria for choosing one modality of re-treatment over the other (re-induction vs. maintenance schedules) varied over time depending on the available evidence in medical literature, but one of the most important factors has been the patient´s vulnerability to relapse, according to the assessment of the expert treating physician.

### Measured outcomes

The main outcomes for HOCF group were the variation in CI and CO medians comparing pre-treatment with post-treatment values (after one complete induction schedule). Secondary outcomes and other follow-up measures included: requirement of bevacizumab re-induction and/or maintenance schedules, hemoglobin levels, orthotopic LT requirement and mortality. Hemodynamic response to treatment was considered complete when CI was normalized (2.5–3.6 ♀/3.9 ♂), partial (decreased but not normalized) or none (if no significant decline). The main outcomes for the IDRA cohort were the change in the medians of: hemoglobin, blood units and ESS, comparing the *baseline* values with respect to the values at 3 months after starting treatment. Secondary outcomes and other follow-up measures for this cohort also included: re-induction requirement with bevacizumab and/or maintenance programs; change in the medians of: hemoglobin, blood units, ESS, iron infusions, hospital admissions for gastrointestinal bleeding and/or epistaxis within a period of 12 months from the beginning of treatment. In addition, the clinical response was defined as the increase in hemoglobin equal to/or above 10 g/dl and at least a fifty percent reduction in the transfusion requirement in the evaluated period. Transfusion response alone was defined as not reaching hemoglobin levels above 10 g/dl, but reducing transfusion requirements in fifty percent or more. Likely or possible adverse events related to bevacizumab treatment for the full cohort were also recorded.

### Statistical analysis

Qualitative variables are presented as proportions. Quantitative variables are presented as median and interquartile range (IQR) or range. Pretreatment and post treatment variations in hemodynamic parameters and pretreatment and three months post treatment variations for hemoglobin, transfusion requirements and ESS were assessed using paired-Wilcoxon Signed Rank test. For subgroup analysis (IDRA patients under anticoagulation therapy and IDRA patients due to epistaxis) pre and three months post treatment variations were compared using paired-Wilcoxon Signed Rank test. All presented p values are two sided. We used a 0.05 threshold to declare statistical significance. The missing data were not imputed. If a data point was missing from one side of a paired-data analysis (baseline values or post-treatment), that “case-patient” was omitted. To asses repeated measures of bleeding indicators over a twelve months period, a longitudinal analysis was conducted. Linear mixed-effects models were fitted for hemoglobin concentration and ESS over time. A Poisson mixed-effects regression was fitted for longitudinal data on transfusion requirement (blood units). Unstructured covariance matrices and Restricted Maximum Likelihood Estimation (REML) were used in all longitudinal models. Wald Test was used to obtain p-values for regression coefficients. We used STATA v. 14.1 for the analysis.

## Results

**Fig 1:**
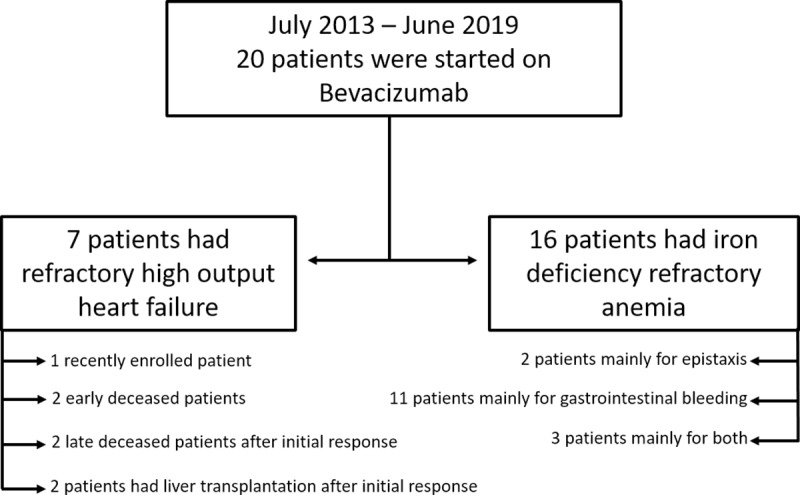
Study flow chart.

The institutional HHT registry has currently 550 patients enrolled with 480 confirmed HHT cases. Between July 2013 the 1^st^ and June the 1^st^ 2019, twenty HHT patients were started on bevacizumab either because of HOCF and/or IDRA and were eligible for entering our observational study (Fig 1). There was only one female patient who could have been a candidate to receive bevacizumab, but due to her high risk of intestinal perforation due to a large previous cecal scar, she was excluded from treatment before starting. There were no treatments with bevacizumab that did not start or ended early due to serious adverse events, lack/revocation of patient consent and/or lack of financial support. Median time of follow up for the whole bevacizumab cohort was 404 [range: 45–2002] days.

Demographic, clinical, analytical and available genetic data from included patients are summarized in Table 1. Median age was 63 [49.5–71] years and 12 (60%) were women. All patients had epistaxis and mucocutaneous telangiectasias, with high prevalence of HVMs and GI compromise (90%), while PAVMs and CAVMS were only present in less than 50% at the cohort. Four patients were started on bevacizumab mainly due to refractory HOCF, 13 patients because of IDRA, and three patients were eligible for both indications.

**Table 1:** Baseline characteristics of study population.

	Full cohort (n = 20)	HOCF (n = 7)	IDRA (n = 16)
Age (years), *median and IQR*	63 [49.5–71]	53 [43–72]	65 [55.5–71]
Female, *n (%)*	12 (60%)	4 (57.1%)	9 (56.3%)
**Genetic testing, *n***			
*ACVRL1*	5		
*ENG*	2		
*MADH4*	0		
Not available	13		
**Disease manifestation, *n (%)***			
Epistaxis,	20 (100%)		
baseline* ESS score, *median and IQR*	1.49 [0.51–4.06]	0.51 [0–3.15]	1.01 [0.26–4.71]
Hospital admissions for gastrointestinal bleeding	11 (55%)	2 (28.6%)	11 (68.8%)
HOCF	7 (35%)	7 (100%)	3 (18.8%)
Encephalopathy	1 (5%)	0	1 (6.3%)
Thrombosis	3 (15%)	0	3 (18.8%)
Atrial fibrillation	6 (30%)	4 (57.1%)	4 (25%)
**Vascular involvement, *n (%)***			
Cutaneous telangiectasia	20 (100%)		
Gastrointestinal telangiectasia	18 (90%)	6 (85.7%)	15 (93.8%)
PAVMs	9 (45%)	2 (28.6%)	7 (43.8%)
HVMs	18 (90%)	7 (100%)	14 (87.5%)
CAVMs	6 (30%)		
**Pro-bleeding factors, *n (%)***			
Antiaggregants	1 (5%)	0	1 (6.3%)
Anticoagulants	5 (25%)	2 (28.6%)	4 (25%)
**Concomitant therapeutic measures at enrollment**			
Diuretics, *n (%)*		7 (100%)	
Beta blockers, Propanolol, *n (%)*		7 (100%), 5 (71.4%)	
Tranexamic acid, *n (%)*			9 (56.3%)
Baseline* blood units requirements, *median and IQR*			4 [2–6]
Baseline* IV iron requirements (mg), *median and IQR*			1400 [1000–2900]
Baseline* minor ENT procedures, *n (%)*			1 (6.3%)
Major ENT procedures, *n (%)*			8 (50%)
Baseline* endoscopic APC procedures, *median and IQR*			0 [0–1]
**Bevacizumab schedule**			
Applications during first year, *median and IQR*	6 [5–6]	6 [2–6]	6 [5–7]
Re-Induction, *n (%)*	6 (30%)	2 (28.6%)	5 (31.3%)
Maintenance, *n (%)*	6 (30%)	1 (14.3%)	6 (37.5%)
**Pre-treatment clinical and echo/cath findings,** *median and IQR*			
Baseline* Pro-bnp (pg/ml)		1797 [1243–3591]	
Cardiac index (l/min/m2)		4.5 [4.1–5.6]	
Cardiac output (l/min)		8.3 [7.5–9.2]	
Baseline* hematologic findings, *median and IQR*			
Hemoglobin (g/dl)	8.35 [7.6–9.4]	9.1 [8–10.4]	8.2 [7.6–9.2]
Ferritin (mg/dl)			93.1 [47.9–137.4]

* Baseline: three month before bevacizumab treatment. HOCF: high output cardiac failure. IDRA: iron deficiency refractory anemia. *ACVRL1*: Activin A Receptor like Type 1. *ENG*: Endoglin. *MDH4*: Mothers against Decapentaplegic Homolog 4. ESS: epistaxis severity score. PAVMs: pulmonary arteriovenous malformations. HVMs: hepatic vascular malformations. CAVMs: central nervous system arteriovenous malformations. ENT: ear-nose-throat. APC: argon plasma coagulation.

### HOCF group results

Seven patients were retrospectively enrolled under this indication. Baseline data is available in detail in Table 1. Median enrollment age was 53 year [43–72] and more than 50% were women. All of patients had HVMs and the median pretreatment CI and CO was 4.5 L/min/m^2^ [4.1–5.6] and 8.3 L/min [7.5–9.2] respectively, with four patients suffering from atrial fibrillation. The whole group presented anemia at baseline with a median pretreatment hemoglobin level of 9.1 g/dl [8–10.4]. These patients were considered refractory to conventional medical therapy, since all of them presented New York Heart Association class III/IV chronic cardiac failure despite adequate hydrosaline restriction and adequate diuretic/beta-blocker therapy. In Table 2 we summarized main hemodynamic parameters in time, as well as their temporal association with bevacizumab applications. We could analyze only four patients who had post-treatment follow-up values. Pre-treatment CI a CO median for this small sample was 5.6 L/min/m^2^ [5.1–5.9] and cardiac output (CO) was 8.6 L/min [8.1–9]. There was a non-significant decline in median post-treatment CI and CO to 4 L/min/m^2^ [3.6–4.2] and to 6.6 L/min [5.6–6.7] respectively (n = 4). Given the heterogeneity between patients, the individual descriptive data is summarized in Table 2.

**Table 2:** High output cardiac failure group summarized data.

		**Baseline**	**3 ms**	**6 ms**	**12 ms**	**18 ms**	**24 ms**	**Comments**
Pt 1: F 47 y/o.	**CI**	5.6	6.3	4.3		4.3		Re-induction after 3 years (bleeding). Died due to a septic shock almost 4 years after enrollment while on LT list
	**Co**	8.6	9.2	6.7		6.7		
	**PSP**		53	44		35		
	**Hb**	9.1	12.1	9.3	9.8	7		
	**Bvz**	**6**					**2**	
		**Baseline**	**3 ms**	**6 ms**	**12 ms**	**18 ms**	**24 ms**	**Comments**
Pt 2: F 53 y/o.	**CI**	4.5			4			Died due to pneumonia about 20 months after enrollment
	**Co**	7.5			6.2			
	**PSP**	45	60		28	45		
	**Hb**	12.4	13.5		12.8	12.5		
	**Bvz**	**6**						
		**Baseline**	**3 ms**	**6 ms**	**12 ms**	**18 ms**	**24 ms**	**Comments**
Pt 3: F 36 y/o.	**CI**	6.2	3.1	3.1			4.9	LT 2 years after enrollment. Alive
	**Co**	9.3	4.6	4.1			7.7	
	**PSP**	36			24		22	
	**Hb**	7	8.7		7.5		7.6	
	**Bvz**	**6**				**3**		
		**Baseline**	**3 ms**	**6 ms**	**12 ms**	**18 ms**	**24 ms**	**Comments**
Pt 4: M 45 y/o	**CI**	4.1		3.4	5.6			LT 1 year after enrollment. Alive
	**Co**	8		6.3	10			
	**PSP**	65	79	121	62			
	**Hb**	9.3	10.7	10.6	8.4	8.5	9.3	
	**Bvz**	**6**						
		**Baseline**	**3 ms**	**6 ms**	**12 ms**	**18 ms**	**24 ms**	**Comments**
Pt 5: F 81 y/o.	**CI**							Died due to cardiogenic shock about 4 days after receiving first dose of bevacizumab
	**Co**							
	**PSP**	72						
	**Hb**	10.4						
	**Bvz**	**1**						
		**Baseline**	**3 ms**	**6 ms**	**12 ms**	**18 ms**	**24 ms**	**Comments**
Pt 6: M 72 y/o	**CI**	4.5						Died due to pneumonia after receiving 2 doses of bevacizumab
	**Co**	9.2						
	**PSP**	71						
	**Hb**	8						
	**Bvz**	**2**						
		**Baseline**	**3 ms**	**6 ms**	**12 ms**	**18 ms**	**24 ms**	**Comments**
Pt 7: M 61 y/o	**CI**	3.8						Alive, recently enrolled
	**Co**	6.7						
	**PSP**	65						
	**Hb**	8.4						
	**Bvz**	**1**						

Pt: patient. M: male. F: female. CI: cardiac index L/m2/min. Co: cardiac output L/min. PSP: pulmonary systolic pressure mmHg. Hb: hemoglobin g/dl. Bvz: number of bevacizumab applications. LT: Liver transplantation.

Patient number one had the longest follow-up time. She had a complete and uneventful bevacizumab induction schedule, but with a late hemodynamic response at about six months after starting treatment. This improvement in CI was sustained in time for almost two years, after what she had a short maintenance schedule when becoming symptomatic. During the third year of follow up, she was started on a re-induction schedule because of GI bleeding. She entered LT list, but unfortunately died because of a septic shock secondary to biliary infection.

Patient number two was started on bevacizumab with good tolerance except for reopening of a previous venous ulcer in her leg. She had a severe tricuspid insufficiency secondary to a previous infective endocarditis in addition to HOCF due to hepatic disease. She improved both clinically and in hemodynamic parameters, but the latters were recorded at twelve months after starting treatment because of medical insurance issues. During the second year of follow up, she was admitted several times with infectious complications and worsening of heart failure and hepatic disease. She was considered not candidate to LT due to her previous cardiac comorbidities and severe pulmonary hypertension. She died before reaching two years of follow-up due to pneumonia.

Patient number three was the youngest. She had great response to bevacizumab induction both echocardiographically and clinically. During the second year of follow-up, she worsened clinically and received a brief maintenance schedule while completing her evaluation for LT. She stopped bevacizumab treatment in waiting list and was successfully transplanted. She is alive and with a good quality of life.

Patient number four had many previous cardiac comorbidities in addition to right overload for HHT-related hepatic disease. He had a prosthetic aortic valve replacement (due to infective endocarditis decades ago) as well as a tricuspid valve plasty and closure of a congenital interventricular communication. He was under anticoagulation therapy both for prosthetic valve and atrial fibrillation, resulting in epistaxis and occult GI bleeding of difficult handling. He was started on bevacizumab for refractory heart failure and improved both clinically and echocardiographically. As an adverse event he developed venous ulcers during treatment. Improvement was only sustained for less than one year. He was evaluated and received a LT with good outcomes.

There were two early deceased patients. Patient number five was an elderly woman with diagnosis of HHT late in life and previous erratic medical contact. She was referred to HHT unit and she was early admitted with an advanced right heart failure and moderate to severe pulmonary hypertension due to severe hepatic disease. She received one application of bevacizumab but continue to worsen and died due to cardiogenic shock four days later. Patient number six had right heart failure and atrial fibrillation, both refractory to medical treatment with diuretics and cardiotonics. He was under anticoagulants due to atrial fibrillation and the presence of an intracavitary atrial thrombus. His anticoagulation tolerance was poor, exhibiting IDRA as a result of hidden GI bleeding. An atrial appendage closure was performed once the thrombus disappeared. To improve heart failure and IDRA, bevacizumab was prescribed, but unfortunately he could only receive two applications (1 month of treatment), since he died because of pneumonia and cardiac failure.

Patient number seven has recently been enrolled and received only one bevacizumab infusion. He has a severe aortic stenosis besides HOCF associated to liver disease and IDRA due to GI bleeding.

### IDRA cohort results

Sixteen patients were retrospectively enrolled under this indication. Baseline data is available in detail in Table 1. Patients were mainly in the 6th and 7th decade of life and more than fifty percent were women. Median *baseline* (3 months before treatment) blood requirement was 4 [2–6] units and median iron requirement was 1400 mg [1000–2900]. Regarding bleeding source, most of our patients had GI telangiectasias with a median total of gastrointestinal endoscopies with APC of 2.5 [range: 0–5]. More than two-thirds of the patients had at least one hospital admission for overt GI bleeding three months before starting bevacizumab. Regarding epistaxis, median ESS score was mild, and more than half of our patients had major ENT procedures performed before enrollment. Main sources of bleeding supporting the bevacizumab prescription were: GI bleeding in eleven, epistaxis in two and both sources in three patients. Almost a quarter of IDRA cohort was under anticoagulation therapy due to atrial fibrillation and/or thromboembolic disease. Induction therapy with bevacizumab was performed in whole cohort at 5 mg/kg with a median number of applications of 5 [2.5–6]. In five patients, re-induction was needed mainly because of deep hemoglobin drop, and six patients needed maintenance therapy due to mild decline in hemoglobin levels in time.

**Fig 2:**
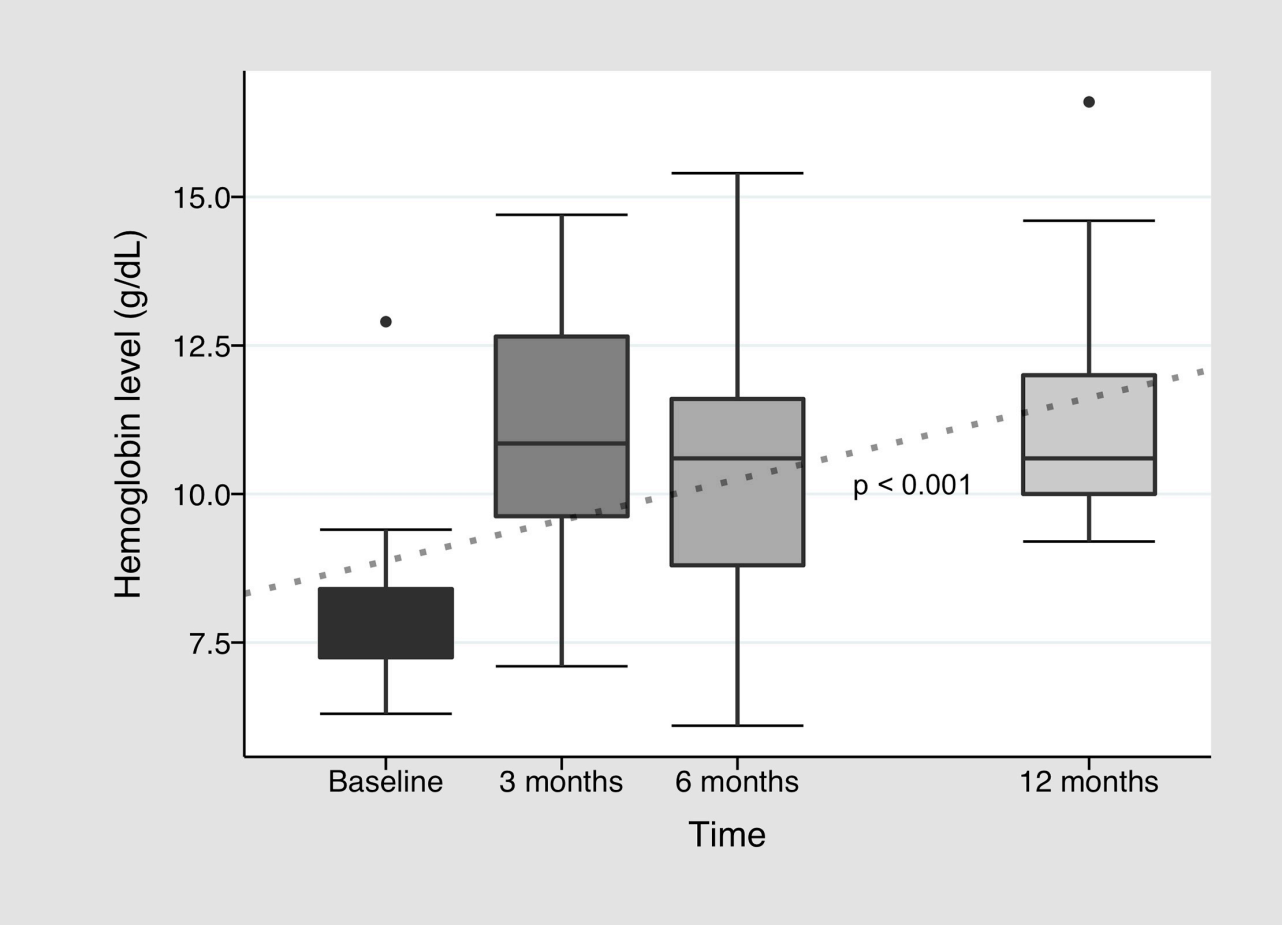
Hemoglobin level variation during bevacizumab treatment. Baseline hemoglobin level represents the summary of all hemoglobin values 3 months before starting bevacizumab treatment. The dotted line is the fitted regression line (Linear mixed effects model).

**Fig 3:**
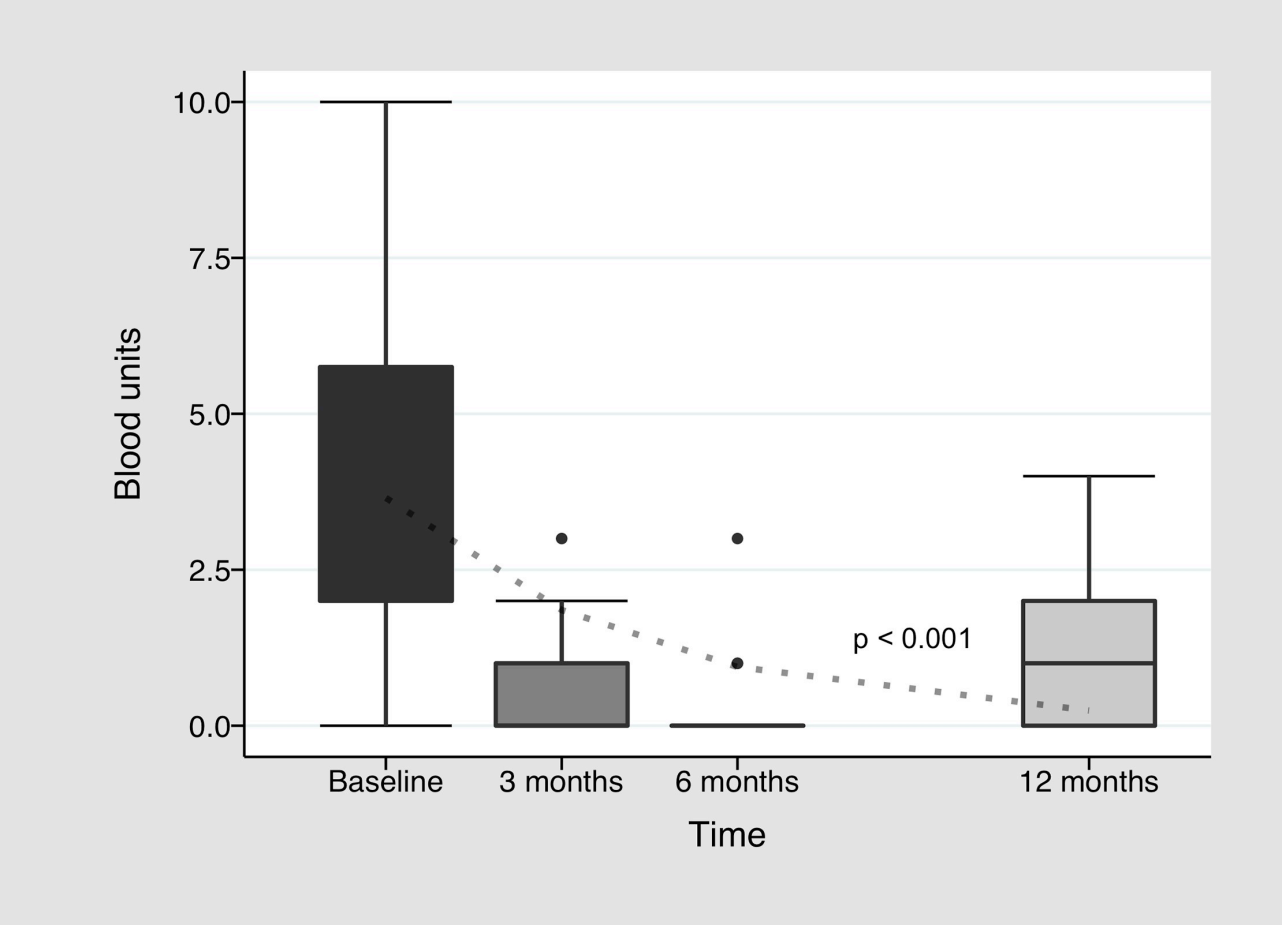
Blood unit requirement during bevacizumab treatment. Baseline blood unit level represents the total number of units received along 3 months before starting bevacizumab treatment. The dotted line is the fitted regression lines (Poisson mixed effects model).

**Fig 4:**
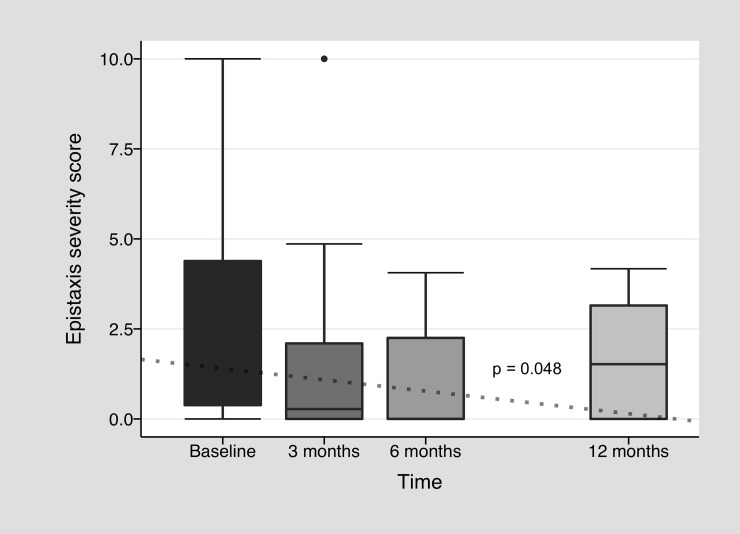
Epistaxis severity score variation during bevacizumab treatment. Baseline score represents epistaxis severity 3 months before starting bevacizumab treatment. The dotted line is the fitted regression line (Linear mixed effects model).

In Fig 2, we outline hemoglobin change during bevacizumab treatment. Improvement in median baseline pretreatment hemoglobin level of 8.1 g/dl [7.2–8.4] was observed all along twelve months after treatment induction with a median level of 10.9 g/dl [9.5–12.8] after three months (*p =* 0.01, n = 14). During this first trimester, there was 64.4% of patients who reached at least 10 g/dl of hemoglobin and reduced their transfusion requirement by at least 50% (clinical response) and 21.4% who did not improved hemoglobin above 10 g/dl, but drastically reduced their transfusion need (transfusion response alone). At six months, median hemoglobin was 10.6 g/dl [8.8–11.6] (n = 13), and clinical response was observed in 61.5%, with 15.4% of transfusion response alone. In the twelfth month, median hemoglobin was 10.6 g/dl [10–12] (n = 9) and 44.4% had clinical response. There was a significant increment in hemoglobin levels all along twelve months (p<0.001). There were also some patients who normalized hemoglobin levels above 14 g/dl (n = 4) in at least one moment during first year after treatment. In line with hemoglobin improvement, a significant decline in blood units requirement was observed, with a median requirement of 0 [0–1] after three months (*p<*0.01, n = 13), 0 [0–0] after six months (n = 13), and to 1 [0–2] after twelve months (n = 9) (Fig 3). This reduction was also significant all along twelve months (p<0.001). There was no significant decrease in iron requirement (see S1 Fig), but 25% of the cohort needed only 1000 mg or less of intravenous iron during follow up. Regarding epistaxis, full IDRA cohort median pretreatment ESS was 1.01 [0.3–4.7], with improvement mostly during first months of treatment: 0.28 [0–2.3] after three months (*p =* 0.01, n = 14), 0 [0–2.3] after six months (n = 13) and 1.52 [0–3.2] after twelve months (n = 9) (Fig 4). Again longitudinal analysis showed a significant reduction of ESS throughout twelve months (p = 0.048). When analyzing only the subgroup of patients who were anemic especially or in part due to epistaxis, median basal ESS was 6.4 [4.1–7.2] (n = 5), improving to 2.2 [1.5–4.9] (*p* = 0.12, n = 5) at three months, to 2.3 [2.3–3.2] (n = 5) at six months and to 3.6 [3.2–4.2] (n = 3) at twelve months. Finally, among patients under anticoagulation therapy, there was no great improvement in hemoglobin levels at 3 months (baseline: 8 g/dl [7.7–10.5] vs. three months: 9.5 g/dl [7.3–12.5]), but there was a clear trend to reduce blood requirement (baseline: 7.5 [3.5–14] vs. 3 months: 2 [0–2]). Each individual patient data in time is available in S2 Fig.

### Adverse events

Bevacizumab treatment was in general well tolerated. Regarding frequent bevacizumab-related toxicities, poor wound healing was observed in four, worsening of previous hypertension was observed in two and asthenia/myalgia in four patients. There was an ischemic non-cardioembolic stroke in one patient during treatment which was not clearly attributable to bevacizumab. In all cases, no adverse event was serious enough for considering stopping treatment. At the time of study analysis, six (30%) patients were dead, most of them in the HOCF group due to severe cardiac failure and/or septic shock (n = 4). Two deaths were recorded under unique IDRA indication, one due to acute myocardial infarction and the other because of contrast induced nephropathy after an aortic valve plasty procedure. In general, deceased patients were older than those who survived, but this difference was not statistically significant.

## Discussion

This observational study outlines that bevacizumab is an effective and well tolerated therapeutic option for treating HHT patients with IDRA, with improvement in hemoglobin levels, epistaxis severity and with reduction in transfusion requirement. It also enforces its use as a bridge therapy for patients with refractory HOCF awaiting for LT.

Improvement in bleeding indicators for IDRA cohort was evident upon the end of induction schedule, around three months after starting treatment as previously reported [26]. This benefit was sustained during the first year without further need for bevacizumab infusions in some cases, but there was a significant proportion of patients with a decline in response between six and twelve months. Those cases needed re-induction schedules, if the worsening was severe, or maintenance schedule for those cases with a slight tendency to worsen. This loss of treatment efficacy over time is also introduced by a French multicenter study with a median time of bevacizumab effect of six months [25]. Despite transfusion decline, there was no significant decrease in iron requirement, but a quarter of the cohort needed 1000 mg or less of intravenous iron during that period. Data showed that few patients also experienced an increase of hemoglobin levels to the upper limit of normality, reaching these surprising levels even with low iron stores. It has been proposed that VEGF may enhance erythropoietin secretion in non-hypoxic states through activation of platelets-derived growth factor receptor (PDGFR) and not through its own receptors [30]. This experimental data make the previous results even more interesting, since bevacizumab would block the effect of increased VEGF before any receptor activation, and it is not clear what would be the role of this VEGF-erythropoietin pathway in chronic anemic patients such as HHT patients.

Data was heterogeneous for HOCF group. Hemodynamic improvement could be observed in almost half of the patients, but that amelioration was dissimilar in between them when considering the strength of the response, time to improvement and duration of the effect. Bevacizumab was useful in half of our analyzable patients, being also those who had the chance to complete at least one induction schedule. Two patients had complete response, and were able to achieve LT while two other patients had partial response but could not achieve it. This hemodynamic improvement has already been outlined by *Dupuis-Girod et al*. in a prospective cohort study of 24 patients in which 3 patients had complete response and 17 patients achieved partial response at three months after starting treatment [24]. Bevacizumab has been proposed as a bridge therapy for patients awaiting LT [31,32] or as a supportive treatment when transplantation is not an alternative or it is rejected by the patient [33]. In the present study, bevacizumab treatment allowed a better clinical performance in two patients awaiting for LT, one of whom was under anticoagulation treatment. As bevacizumab may delay wound healing, it is advised to allow at least one month of break in between infusion of this drug and a surgical procedure. This issue must be taken into account in patients ranking high on LT list. At this time there is uncertainty about the ideal time frame for using bevacizumab in this population, especially in those in whom LT is a plausible therapeutic option [23].

There is uncertainty about predictors for the bevacizumab response among patients, considering not only the magnitude of the effect but also its sustainability over time. We could raise the hypothesis that advanced age; severely affected patients such as those with persistent epistaxis despite advanced clinical/surgical approach, severe involvement of GI tract and/or long lasting severe liver disease; and those under anticoagulation therapy, could achieve weaker and shorter responses. In the present study, patients with the worst outcome (death) tended to be older. We did not find a significant improvement in hemoglobin levels in patients under anticoagulation therapy, except for a reduction in transfusion requirement, but both results should be confirmed in larger studies. It could be argued that the aforementioned lack of hemoglobin improvement but reduction in transfusion requirement, could be suggesting a reduction in the number of overt bleeding events, even though these patients may still have chronic persistent occult bleeding perpetuating iron deficiency. In addition, those patients with IDRA due mostly to epistaxis, experienced clinical improvement when considering ESS in time, but this was not statistically significant and there was no significant improvement in hemoglobin levels. These results differ from those found by *Iyer et al* [26]. However, it is important to highlight, that this is a very small sample of a population of patients with refractory epistaxis. In our center, we usually prefer exhausting all pharmacologic, minor and major ENT procedures before considering bevacizumab. On the other hand, some patients with chronic GI bleeding have multiple, diffuse and large recurrent telangiectasias, and in those cases we do not usually attempt to perform repetitive endoscopic treatments. Moreover, we have previously reported that in some patients, APC treatment might act as a “second hit” and trigger abnormal angiogenesis with development of raised bleeding injuries [34]. In this scenario of extensive GI compromise, bevacizumab arises as a promising therapeutic option.

Although the study has weaknesses derived from its observational nature, it strengthens the body of evidence in favor for the use of bevacizumab. As many other published observational studies, it was not possible to perform a multivariate analysis to assess the real impact of bevacizumab on the results, due to the lack of matched controls or randomization. A longitudinal analysis was performed for paired data for the IDRA cohort. It is important to note that the dotted lines observed in Figs 2, 3 and 4 represent the behavior of hemoglobin, blood units and ESS over time and how different this behavior is compared to a hypothetical patient without treatment (no changes in pretreatment values over time). The behavior of bleeding parameters should be approached with caution, since each analysis was not adjusted for confounding variables and does not take into account the effect of the bevacizumab re-treatment over time. Despite this, the improvement in hemoglobin and ESS medians, and the decline in transfusion requirements, were all in line with the bevacizumab hypothesis of efficacy. Finally, regarding HOCF group, sample size was not powerful enough for dropping significant results, but it remains a detailed and pragmatic descriptive outline of a single center institutional registry.

Small studies such as this one, cannot assess the safety of bevacizumab treatment but only describe good tolerance among included patients. Of the study deceased patients, severity of the underlying disease may explain the adverse outcome better than exposure to bevacizumab treatment, but more studies that focus on toxicity are needed [35]. In this regard, half of the patients who died in this cohort had septic shock (starting from pneumonia/cholangitis) as a cause of death. A small increase in the risk of infections has been reported in patients treated with bevacizumab, but HHT may also predispose to an increased risk of infections, given the reported involvement of endoglin in macrophage migration processes through the endothelium in proinflammatory states [36]. Finally, although bevacizumab could represent a useful and safe treatment for selected patients, its high cost and availability could represent a limiting factor for its wide use. Future studies focusing on cost-effectivity are also required.

In conclusion, bevacizumab is a promising therapeutic strategy for HHT patients with refractory HOCF awaiting LT or when the latter is not an alternative. Regarding IDRA patients, especially due to GI involvement, bevacizumab seems a reasonable and effective option for improving anemia. Caution should be exercised by assessing the balance between effectiveness and safety in patients with less severe forms of anemia but with intermittent response to conventional treatment until we have better evidence from multicenter studies.

## Supporting information

S1 Fig Intravenous iron requirements during bevacizumab treatment. Baseline iron represents the total mg of iron received along three months before starting bevacizumab treatment.
(PNG)

S2 Fig Iron deficiency refractory anemia cohort summarized data. Behavior of hemoglobin levels and transfusion requirements for each enrolled patient in IDRA cohort. Y axis shows individual patient numerical order. Continuous solid line shows hemoglobin trend for twelve months or until data is available (no hemoglobin numerical data on the Y axis) and requirement of blood units (in circulated numbers), during each period of time (indicated on the X axis). When no significant response was observed over time, a black line is shown as opposed to a gray response line.
(PNG)
