# Supplementary figures and images for: Bevacizumab for treating Hereditary Hemorrhagic Telangiectasia patients with severe hepatic involvement or refractory anemia
Source: PLoS One. 2020 Feb 7;15(2):e0228486. doi: 10.1371/journal.pone.0228486 (PMC7006931; doi:10.1371/journal.pone.0228486)

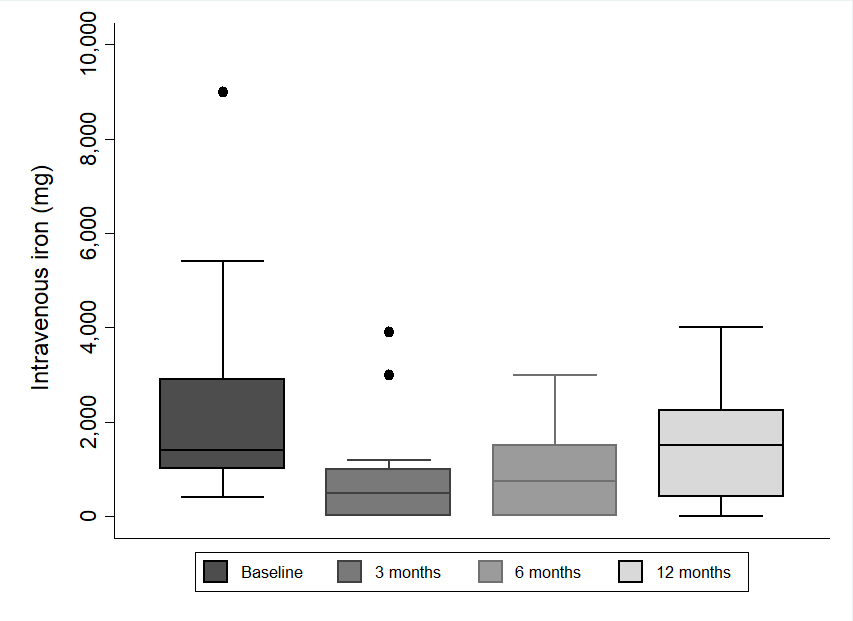

Supplement: S1 Fig — Baseline iron represents the total mg of iron received along three months before starting bevacizumab treatment. (PNG) [file pone.0228486.s001.png]

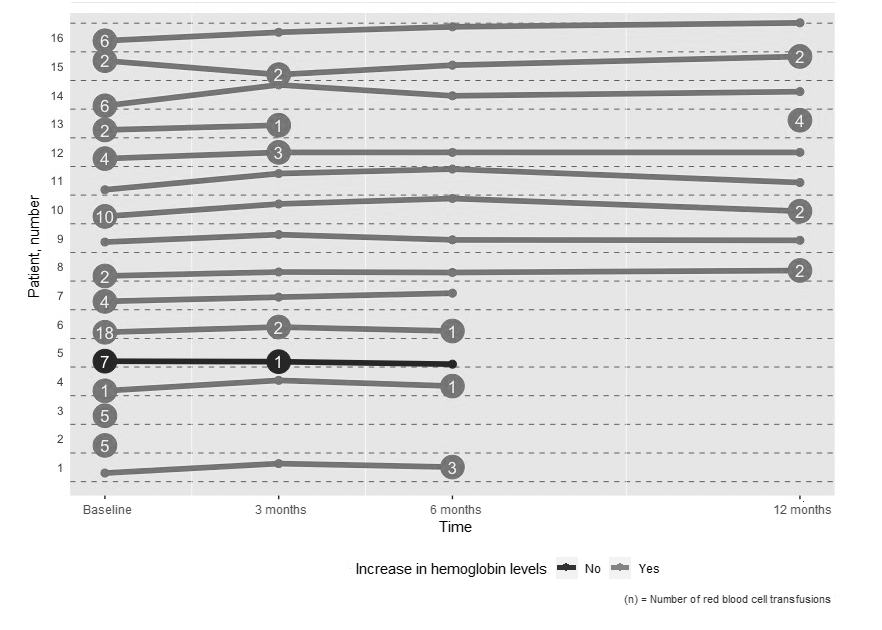

Supplement: S2 Fig — Behavior of hemoglobin levels and transfusion requirements for each enrolled patient in IDRA cohort. Y axis shows individual patient numerical order. Continuous solid line shows hemoglobin trend for twelve months or until data is available (no hemoglobin numerical data on the Y axis) and requirement of blood units (in circulated numbers), during each period of time (indicated on the X axis). When no significant response was observed over time, a black line is shown as opposed to a gray response line. (PNG) [file pone.0228486.s002.png]
